# Coagulation Tests and Reversal Agents in Patients Treated with Oral Anticoagulants: The Challenging Scenarios of Life-Threatening Bleeding and Unplanned Invasive Procedures

**DOI:** 10.3390/jcm13092451

**Published:** 2024-04-23

**Authors:** Andrea Pozzi, Fabiana Lucà, Sandro Gelsomino, Maurizio Giuseppe Abrignani, Simona Giubilato, Stefania Angela Di Fusco, Carmelo Massimiliano Rao, Stefano Cornara, Giorgio Caretta, Roberto Ceravolo, Iris Parrini, Giovanna Geraci, Carmine Riccio, Massimo Grimaldi, Furio Colivicchi, Fabrizio Oliva, Michele Massimo Gulizia

**Affiliations:** 1 Cardiology Division Valduce Hospital, 22100 Como, Italy; andreawellsvabg@gmail.com; 2 Cardiology Department, Grande Ospedale Metropolitano, GOM, AO Bianchi Melacrino Morelli, 89129 Reggio Calabria, Italymassimo.rao@libero.it (C.M.R.); 3 Cardiothoracic Department, Maastricht University Hospital, 6229 HX Maastricht, The Netherlands; 4 Operative Unit of Cardiology, P. Borsellino Hospital, 91025 Marsala, Italy; maur.abri60@gmail.com; 5 Cardiology Department, Cannizzaro Hospital, 95126 Catania, Italy; simogiub@hotmail.com; 6 Clinical and Rehabilitation Cardiology Department, San Filippo Neri Hospital, ASL Roma 1, 00135 Roma, Italy; doctstefania@hotmail.com (S.A.D.F.); furio.colivicchi@gmail.com (F.C.); 7 Arrhytmia Unit, Division of Cardiology, Ospedale San Paolo, Azienda Sanitaria Locale 2, 17100 Savona, Italy; stefano.cornara@gmail.com; 8 Sant’Andrea Hospital, ASL 5 Regione Liguria, 19124 La Spezia, Italy; giorgio.caretta@gmail.com; 9 Cardiology Unit, Giovanni Paolo II Hospital, 97100 Lamezia, Italy; roberto_ceravolo@yahoo.it; 10 Cardiology Department, Mauriziano Hospital, 10128 Torino, Italy; irisparrini@libero.it; 11 Cardiology Unit, S. Antonio Abate Hospital, ASP Trapani, 91016 Erice, Italy; giovannageraci@hotmail.com; 12 Cardiovascular Department, Sant’Anna e San Sebastiano Hospital, 81100 Caserta, Italy; carminericcio8@gmail.com; 13 Department of Cardiology, General Regional Hospital “F. Miulli”, 70021 Bari, Italy; m.grimaldi@miulli.it; 14 Cardiology Unit, ASST Grande Ospedale Metropolitano Niguarda, 20162 Milano, Italy; fabri.oliva@gmail.com; 15 Cardiology Department, Garibaldi Nesima Hospital, 95122 Catania, Italy

**Keywords:** reversal agents, atrial fibrillation, DOACs

## Abstract

In clinical practice, the number of patients treated with direct oral anticoagulants (DOACs) has consistently increased over the years. Since anticoagulant therapy has been associated with an annual incidence of major bleeding (MB) events of approximately 2% to 3.5%, it is of paramount importance to understand how to manage anticoagulated patients with major or life-threatening bleeding. A considerable number of these patients’ conditions necessitate hospitalization, and the administration of reversal agents may be imperative to manage and control bleeding episodes effectively. Importantly, effective strategies for reversing the anticoagulant effects of DOACs have been well recognized. Specifically, idarucizumab has obtained regulatory approval for the reversal of dabigatran, and andexanet alfa has recently been approved for reversing the effects of apixaban or rivaroxaban in patients experiencing life-threatening or uncontrolled bleeding events. Moreover, continuous endeavors are being made to develop supplementary reversal agents. In emergency scenarios where specific reversal agents might not be accessible, non-specific hemostatic agents such as prothrombin complex concentrate can be utilized to neutralize the anticoagulant effects of DOACs. However, it is paramount to emphasize that specific reversal agents, characterized by their efficacy and safety, should be the preferred choice when suitable. Moreover, it is worth noting that adherence to the guidelines for the reversal agents is poor, and there is a notable gap between international recommendations and actual clinical practices in this regard. This narrative review aims to provide physicians with a practical approach to managing specific reversal agents.

## 1. Introduction

It has been reported that approximately 1–2% of the population needs long-term anticoagulation therapy [1]. Due to the safer profile of direct oral anticoagulants (DOACs) when compared to vitamin K antagonists (VKA) [2,3,4,5], the prevalence of patients receiving DOACs has risen in recent decades, encompassing approximately 68 to 79% of the total anticoagulated population in Europe and the United States [6,7,8].

Although hemorrhagic complications are less likely to occur in DOAC patients than in those on VKAs, potential bleeding and unplanned surgical procedures still pose challenging clinical scenarios for patients undergoing anticoagulant therapy [9,10,11]. Of note, timely management of these patients is of paramount importance during the acute phase [12]. In the case of life-threatening bleeding or urgent surgical procedures, the tempestive discontinuation of DOACs is needed [12].

Furthermore, clinicians should thoroughly understand the strategies required to evaluate coagulation status and proficiently utilize different tests to measure the activity of each DOAC [12].

Additionally, administering procoagulants and/or specific antidotes may be necessary in the acute phase. Some procoagulant agents, especially four-factor prothrombin complex concentrates (4PCCs), have been associated with successful treatment of bleeding but may, on the other hand, increase thrombotic risk [13]. Recently, two specific reversal drugs, idarucizumab (dabigatran) and andexanet (factor Xainhibitors), were shown to neutralize the anticoagulant effects of DOACs. Nevertheless, to date, 4PCCs have not been compared to specific reversal agents in randomized controlled trials (RCTs) [14,15].

This review aims to describe different coagulation tests and reversal agentsthat may be used in critical scenarios according to the specific anticoagulant regimens (VKAs, thrombin inhibitors, or Xa inhibitors), focusing on the management of life-threatening bleeding and unplanned surgical procedures. We also present epidemiological data regarding the incidence of major bleeding events in patients undergoing OAC therapy, comparing data between patients on DOACs and those treated with VKAs.

### 1.1. Coagulation Tests

Screening coagulation tests, such as prothrombin time (PT), activated partial thromboplastin time (aPTT), and thrombin time (TT), may offer only limited information in patients undergoing DOAC treatment. In contrast, they are commonly used in patients on VKA or unfractionated heparin (UFH) [16,17,18]. Table 1 reports the impact of DOAC therapy on different coagulation tests.

### 1.2. Activated Partial Thromboplastin Time (aPTT) 

The aPTT test assesses the functionality of the intrinsic coagulation pathways and tends to be prolonged in patients treated with dabigatran. The dabigatran dose–response curve with aPTT reagents is non-linear but curvilinear, flattening at high dabigatran levels. The aPTT test cannot be used to monitor the anticoagulation effect of dabigatran.Nevertheless, it could provide an approximate assessment of the anticoagulant activity of dabigatran. Depending on reagent sensitivity, aPTT provides only qualitative or semi-quantitative results in patients treated with dabigatran.

### 1.3. Specific Coagulation Tests in Patients Treated with Dabigatran

Thrombin time (TT) is the most sensitive test for dabigatran anticoagulant activity [19,20]. This test measures the clot formation time by adding high-sensitivity thrombin to plasma. Indeed, a prolonged thrombin time can also be occur in the presence of a very low dabigatran concentration with a high negative predictive value. Hence, in the presence of a normal TT in patients with life-threatening bleeding or unplanned invasive procedures, this test excludes the presence of a relevant concentration of dabigatran. However, two important pitfalls are related to the standardized amount of thrombin used in the test assay and the presence of different available assays [19]. 

Another test used to measure the concentration of dabigatran is the diluted thrombin time. This test has been demonstrated to be less sensitive to dabigatran anticoagulant activity than TT. The level of dabigatran is determined using a calibration curve constructed with calibrants containing a known concentration of dabigatran [21]. In the presence of a value > 30 ng/mL, the use of a specific dabigatran reversal agent should be considered [18].

Finally, the ecarin clot time (ECT) is a meizothrombin test that allows precise quantification of dabigatran [22]. Ecarin is a snake venom able to convert prothrombin into meizothrombin, a prothrombin–thrombin intermediate.Direct thrombin inhibitors interfere with this activity. Similarly to the diluted thrombin time test, this test provides the dabigatran concentration measurement by comparing the clotting time with that in a calibration curve constructed with samples containing a known amount of dabigatran. If the level exceeds 30 ng/mL, the use of a reversal agent should be considered [18].

### 1.4. Coagulation Test in Patients Treated with XaInhibitors

**Figure 1:**
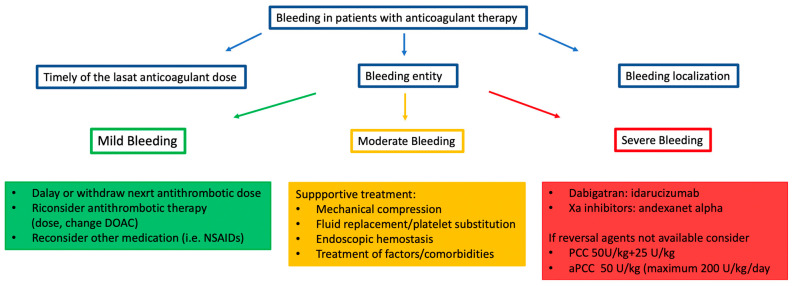
Perioperative bleeding management in patients with anticoagulation therapy. Legend: DOAC: direct oral anticoagulant; NSAID: nonsteroidal anti-inflammatory drug; PCC: prothrombin complex concentrate; aPCC: activated prothrombin complex concentrate; U: unit; Kg: kilogram.

The plasma levels of rivaroxaban, apixaban, and edoxaban are assessed using the calibrated chromogenic anti-Xa assay [23]. The test involves adding factor Xa to plasma containing chromogenic Xa-directed substrate. The Xa inhibitors reduce the color change according to the drug’s blood concentration. By comparing the result with the standard curves constructed with samples containing known concentrations of rivaroxaban, apixaban, and edoxaban, this test allows the quantification of Xainhibitor levels. If the level exceeds 30 ng/mL, the use of a reversal agent should be considered [12,24]. (Figure 1).

### 1.5. Complications Related to Bleeding Associated with Anticoagulant Therapy

Intracranial bleeding, or intracranial hemorrhage (ICH), is a widely acknowledged complication associated with the use of OACs and antiplatelet therapy (APT) [25].

A seven-fold to ten-fold higher risk of intracerebral hemorrhage (ICH) has been observed among patients receiving oral OACs compared to the general population, with mortality rates ranging from 40% to 65% within 30 to 90 days [26,27,28,29,30]. 

A population study indicated that approximately 20% of individuals with ICH were taking OACs [31]. In real-world scenarios, there has been an increase in ICH reports associated with DOACs [32,33,34,35,36].

The prognosis for ICH associated with OACs is typically severe, with up to three-quarters of patients either dying or experiencing long-term disability [32,37]. In patients receiving OACs, ICHs tend to be larger and more commonly result in hematoma volume expansion [37]. In the Tromsø Study, the utilization of OACs has been linked to increased mortality rates related to ICH [38]. The data concerning the prognostic impact of hematoma expansion or bleeding volumes in patients treated with DOACs or VKAs are heterogeneous [39,40,41,42,43,44]. Any difference in ICH volume, 90-day rate of hematoma expansion, or all-cause mortality between DOACs or VKAs has been shown [39]. Other studies reported a comparable hematoma volume expansion between the two treatments with no difference in ICH-related neurologic damage [40,41]. However,, different studies supported the presence of smaller ICHs among patients treated with DOACs compared to VKA. Specifically, Kurogi et al. demonstrated a significantly lower likelihood of moderate or severe consciousness impairment among patients with DOAC-associated ICHs compared to VKA-associated ICHs in more than 2000 patients with ICH. Additionally, the need for surgical removal and death were also reduced among patients treated with DOACs. Different studies have largely demonstrated the reduction in in-hospital ICH-related mortality among patients receiving treatment with DOACs [42,43] (Table 2).

Since ICH in anticoagulated patients represents a life-threatening scenario, the development of a tool capable of promptly identifying anticoagulated patients at heightened risk of experiencing ICH could be of paramount importance [44]. In this context, Park et al. performed the Hemorrhage Estimate Risk in Oral Anticoagulation for Mild Head Trauma (HERO-M) score on a cohort comprising 1425 patients receiving DOACs [45]. This score was effective in identifying patients with an increased risk of ICH. Specifically, previous neurosurgery, post-traumatic amnesia, post-traumatic vomiting, post-traumatic loss of consciousness, and trauma above the clavicles were variables associated with an increased odds ratio of ICH [46].

Although specific studies to assess the management of anticoagulated patients with ICH are unavailable, the Swiss Society of Hematology has recently provided some recommendations [44,46,47]. Specifically, in the presence of ICH, andexanet alfa has been suggested in those patients without severe neurological impairment (Glasgow Coma Scale > 7), with symptom onset occurring in less than sixh, life expectancy >1 month, and last intake of apixaban/rivaroxaban < 15 h or anti-Xa >100 ng/mL. Since there is a lack of data available from the ANNEXA-4 trial regarding patients treated with edoxaban [15], the Swiss Society of Hematology does not consider the use of andexanet alfa but rather four-factor PCC in patients treated with these Xa inhibitors [47]. However, a recent study assessed the efficacy of andexanet alfa in significantly reducing factor Xa activity in 36 patients treated with edoxaban [48].

Hence, patients should receive counseling that emphasizes the importance of achieving a delicate equilibrium between effective antithrombotic prophylaxis and ensuring a safe drug profile [49].

**Table 2:** Comparison between DOAC and VKA in different clinical scenarios.

Clinical Scenario	DOACs vs. VKAs
ICH	- DOACs—lower prevalence of moderate/severe consciousness impairment [42,43]. - Lower risk of death and surgical ICH removal with DOACs [43,50]
GIB	- Rivaroxaban—higher risk of GIB compared to warfarin but no difference in fatal bleeding [3] - Apixaban—lower risk compared to warfarin [4] - Edoxaban, 60 mg—comparable risk to warfarin [5] - Edoxaban, 30 mg—lower risk compared to warfarin [5] - Dabigatran, 150 mg—increased risk compared to warfarin [2] - Dabidatran, 110 mg—comparable risk to warfarin [2] - Observational study: reduced risk of hospitalization or transfusion with DOACs compared to VKAs [51]
Perioperative bleeding	Lower risk of perioperative bleeding with DOACs compared to VKA [52].

Legend: ICH: intracranial hemorrhage; GIB: gastrointestinal bleeding; mg: milligram; DOACs: direct oral anticoagulants; VKA: vitamin K antagonists.

### 1.6. Gastrointestinal Bleeding

Gastrointestinal bleeding (GIB) represents a significant adverse event in patients undergoing treatment with antithrombotic therapies.

OAC therapy increases the risk of bleeding, especially in the presence of gastrointestinal lesions [53]. 

Various mechanisms through which anticoagulant agents might contribute to gastrointestinal (GI) bleeding have been proposed.

Direct factor Xa inhibitors are active agents and are not entirely absorbed, potentially leading to a direct topical impact on GI tissues, thereby elevating the risk of bleeding [54]. The prodrug dabigatran demonstrates a 6% oral bioavailability, and the unabsorbed prodrug may undergo intraluminal activation during passage through the GI tract [54]. Factor Xa inhibitors exhibit higher oral bioavailability rates (50–80%) compared to dabigatran and are noted to possess distinct GI bleeding safety profiles [54].

Another potential mechanism by which anticoagulants may induce bleeding is compromising the GI mucosa’s integrity [54].

In the RE-LY trial, the risk of bleeding was increased in patients treated with 150 mg of dabigatran compared to warfarin. In contrast, no significant difference was reported between 110 mg of dabigatran bid and warfarin [2]. Interestingly, a sub-analysis of the RE-LY trial, using the European label for dabigatran administration, showed a comparable risk in terms of major GIB between dabigatran and warfarin [55].

Conversely, in the ROCKET-AF trial, patients treated with rivaroxaban experienced a higher risk of GIB than those in the warfarin arm. However, no difference in fatal bleeding was shown [3]. In the ENGAGE AF-TIMI 48 study, the risk of GIB was slightly increased when edoxaban 60 mg was compared to warfarin, whereas the risk was similar between edoxaban 30 mg and warfarin [5]. Unlike the aforementioned DOACs, apixaban seems to be the only DOAC that caused no increased risk in GIB compared to warfarin in the ARISTOTELE trial [4] (Table 2).

In clinical scenarios, DOACs have been associated with a safer profile characterized by a lower risk of bleeding than warfarin, although some differences have been highlighted between DOACs [44]. In this regard, Holster et al., in another meta-analysis, reported a consistently higher risk of GIB among patients receiving dabigatran and rivaroxaban but not among those receiving apixaban and edoxaban. However, it is important to highlight that this meta-analysis included heterogeneous groups of patients with AF, venous thrombosis, and thromboprophylaxis following orthopedic surgery [56].

Beyond a lower risk of GIB, findings from a retrospective study suggest that treatment with DOACsis is also associated with a reduced risk of hospitalization and transfusion compared to other OACs [51].

Interestingly, the site of GIB may vary depending on the specific DOAC being used [53]. A higher risk of lower GIB has been reported in subjects on dabigatran [53,57], whereas rivaroxaban has been associated with upper and lower gastrointestinal bleeding rates of 76% and 24%, respectively. Conversely, GB associated with edoxaban exhibited an equal prevalence in both the upper and lower gastrointestinal tract [58].

Among patients treated with VKA, the presence of cardiovascular (CV) and chronic kidney disease, liver cirrhosis, age >65 years, previous GIB, and previous stroke have been associated with an increased risk of GIB [59,60,61].

### 1.7. Perioperative Bleeding and Anticoagulant Therapy

In addition to the risks of bleeding associated with a specific surgical procedure, it is crucial to evaluate patient-related factors that may elevate the risk of bleeding. 

The assessment of patient-related risk involves factors such as the patient’s age, the presence or absence of CV risk factors, established CV disease, comorbidities, a history of prior bleeding complications (especially within the preceding three months), occurrence of bleeding during similar procedures, platelet dysfunction, concurrent use of antiplatelet therapy, and the presence of trauma.

During the perioperative phase, patients receiving OACs should be evaluated, balancing the risks of bleeding and thromboembolism. In this context, both the option of delaying surgery and of ensuring hemostasis may cause challenging consequences.

Nearly 25% of patients undergoing treatment with anticoagulants require a temporary cessation of either DOACs or VKAs for planned surgery [62].

The administration of OACs in the context of neuraxial anesthesia elevates the risk of spinal or epidural hematoma, which can have dangerous consequences. According to international guidelines [63], DOACs should be discontinued before neuraxial procedures, with recommendations ranging from 4 to 5 days for dabigatran and 3 to 5 days for factor Xa inhibitors. The resumption of the anticoagulant is advisable 24 hours post-procedure [64]. In 7201 patients undergoing urgent or emergent coronary artery bypass grafts (CABGs), no notable differences were detected in terms of minor bleeding, major bleeding (MB), or mortality among those who were treated with a DOAC within five days before surgery [65].

Among patients who underwent proctological surgery, those receiving antithrombotic therapy demonstrated a heightened incidence of secondary bleeding compared to controls. Notably, patients on DOACs exhibited the most severe bleeding [66].

Data from a meta-analysis comprising 14 studies involving patients on VKAs, DOACs, or no anticoagulant therapy who underwent hip fracture surgery revealed that performing hip fracture surgery within 48 of DOAC-treated patients has also been considered safe, with a small increase in blood transfusion risk [67] (Table 2).

In a study investigating the likelihood of peri-procedural bleeding following polypectomy among patients receiving treatment with warfarin and DOACs, compared to those without antithrombotic treatment, the prevalence of periprocedural bleeding was higher among patients treated with OAC compared to the control. Notably, the risk of post-polypectomy bleeding was higher among those treated with VKA, demonstrating the safer profile of DOAC treatment (Table 2) [52].

Interestingly, the GIHP-NACO registry enrolled 478 patients treated with DOACs and hospitalized for urgent procedural interventions. This study was performed before DOAC reversal agents became available and demonstrated that invasive procedures were delayed in nearly half of the cases, reducing the bleeding risk. Of note, the rate of excessive bleeding was low, suggesting that most urgent procedures can be safely performed without the use of reversal agents [68]. Contrasting results were shown in another systematic review and meta-analysis. Shah et al. showed an increased risk of bleeding among patients continuing DOACs compared with a DOAC interruption strategy 1 to 4 days before surgery in some but not all studies. The authors concluded that there is no certain evidence supporting the interruption of DOACs versus continuation in the perioperative period. Importantly, this meta-analysis also demonstrated that DOAC interruption followed by a bridge therapy with low-molecular-weight heparin may be associated with a significantly increased risk of bleeding [69].

Interestingly, the PAUSE study identified a standardized perioperative management strategy during elective surgery or procedure for patients with AF who were taking a DOAC, considering DOAC type, renal function, and surgery/procedure-related bleeding risk. The PAUSE protocol was demonstrated to be safe and easily applied for each DOAC to manage both pre-procedure interruption and post-procedure resumption [70].

Dental implantation represents another challenging scenario among patients treated with OACs.

A prospective study evaluating the prevalence and severity of bleeding following dental surgery in patients both with and without anticoagulation treatment revealed an increase in postoperative bleeding among those who underwent dental surgery on OAC. Nevertheless, the severity of bleeding remained low [71]. A meta-analysis revealed no statistically significant difference in the risk of bleeding between OAC therapy and the control group. However, there was a non-significant trend toward an elevated risk of bleeding associated with VKA, whereas no increased risk has been shown among patients treated with DOACs [72]. 

Another meta-analysis comparing the incidence of bleeding in patients receiving uninterrupted DOACs or VKAs who underwent dental extraction suggested a lower risk of bleeding among patients undergoing DOAC therapy. However, the conclusions of this meta-analysis must be taken with caution since the data came from very low-quality evidence with scarce data on each DOAC [73].

In this regard, the GIHP-NACO registry, conducted on 478 patients hospitalized for urgent procedural interventions in therapy with dabigatran, rivaroxaban, or apixaban before specific antidotes became available, showed that nearly half of those who were treated with DOACs undergoing urgent invasive procedures experienced delays and had a lower incidence of excessive bleeding. These data suggest that most urgent procedures might be performed safely without the need for DOAC reversal [68].

The PAUSE study identified, among patients with AF who were taking a DOAC, a standardized perioperative management strategy during elective surgery or procedure considering DOAC type, renal function, and surgery/procedure-related bleeding risk. The PAUSE protocol was demonstrated to be safe and easily applied for each DOAC to manage both pre-procedure interruption and post-procedure resumption [70].

Dose regimen, renal function, and patient’s age did not determine a difference in bleeding or thromboembolic events [74,75] (Figure 1). In patients requiring high-bleed-risk procedures (i.e., cardiac, intracranial, aortic aneurysm surgery), the rate of the bleeding events was significantly higher than in patients undergoing low-bleed-risk procedures (pacemaker implantation, tooth extraction) in patients on apixaban (2.9 vs. 0.59%; *p*< 0.01); no difference was found in patients treated with dabigatran or rivaroxaban. The bleeding risk related to the procedure did not influence the risk of thromboembolism [76].

Despite the availability of clinical guidelines about the periprocedural interruption of DOACs specifying timing according to not only their pharmacodynamics and pharmacokinetics but also the type of planned surgery and renal function, the available evidence concerning the perioperative management of OACs still needs to There is a lack of high-quality studies to support the daily practice of bridging with heparin during the disruption of OACs in patients undergoing an elective procedure (or surgery), and also, no high-quality evidence is available for the interruption of VKA therapy for minor procedures (cardiac device implantation included) or for the continuation or short-term suspension of a DOAC in the perioperative period [69]. There are also data on real-world practices compared to existing clinical practice guidelines for the reversal of OACs before emergency surgery. Data from a survey revealed that merely 32% of institutions had established protocols for emergency anticoagulant reversal. This underscores the need for institutions to formulate guidelines that draw from the recommendations of clinical practices and management algorithms, incorporating insights from healthcare professionals routinely engaged in the care of these patients [77].

Given the high prevalence of the use of DOACs, the possibility of encountering patients who need urgent surgery or have undergone life-threatening bleeding is constantly increasing [78] and effective strategies to manage these conditions are limited [79]. Furthermore, challenges for the emergency physician as well as for all healthcare providers result from the increasing age of OAC patients, the difficulties in establishing the last intake of OAC type and dosage, interpreting coagulation test results in emergencies, and making decisions for or against OAC reversal strategies in acute bleeding or urgent surgery [80]. Managing this peculiar subset of patients is challenging; well-coordinated interdisciplinary teamwork is required [62]. Prothrombin activation time, partial thromboplastin time, DOAC plasmatic level, or other specific coagulation tests are not commonly available, and many physicians are unaware of their use [62]. Fortunately, idarucizumab (an antibody that reverses the effects of dabigatran) has now been approved, and another reversal agent, andexanet alfa, a factor Xa decoy, has been approved as an antidote to inhibitor Xabans (edoxaban, apixaban, and rivaroxaban) [81]. 

### 1.8. Practical Management Strategy for Patients Undergoing Unplanned Surgery or ExperiencingMajor Bleeding

The peri-operative management of patients with anticoagulant therapy is a daily problem faced in emergency rooms worldwide. The main factors that could influence patient management are the urgency of the procedure and the related bleeding risk [82] (see Figure 1).

When patients need non-deferrable major surgery, it is often not possible to VKA or DOAC 6 days before or 24–72 hours before discontinuation of anticoagulant therapy (DOAC) [83]. 

The bleeding and thrombotic risks associated with both the procedure and patient characteristics should be carefully considered [84]. When the thrombotic risk is very high, a full drug reversal should be considered only in the presence of life-threatening bleeding (83). Each surgery presents a different bleeding risk [85] that should be carefully assessed and matched with the patient’s unique profile. Despite several recommendations and documents [86,87,88], the balance between ischemic and bleeding risk is not always straightforward, and different variables may increase the challenges. Indeed, in several situations, a single reversal agent could not be enough in the acute phase, and, on the other hand, a prothrombotic rebound might also occur after the reversal. To add complexity, the bleeding risk is further increased if the mechanical compression of the intervention site is unfeasible [82]. Different issues may also arise to assess whether and when an early resumption of anticoagulation treatment is necessary. Since there are more questions than answers on this important topic, an interdisciplinary team is mandatory to discuss the advantages and disadvantages of therapeutic options.

The primary challenge in managing MB is the absence of a universally accepted definition.

The American College of Cardiology 2020 expert consensus [12] suggests updating and simplifying the definition of MB, especially in patients with antithrombotic drugs.

**Figure 2:**
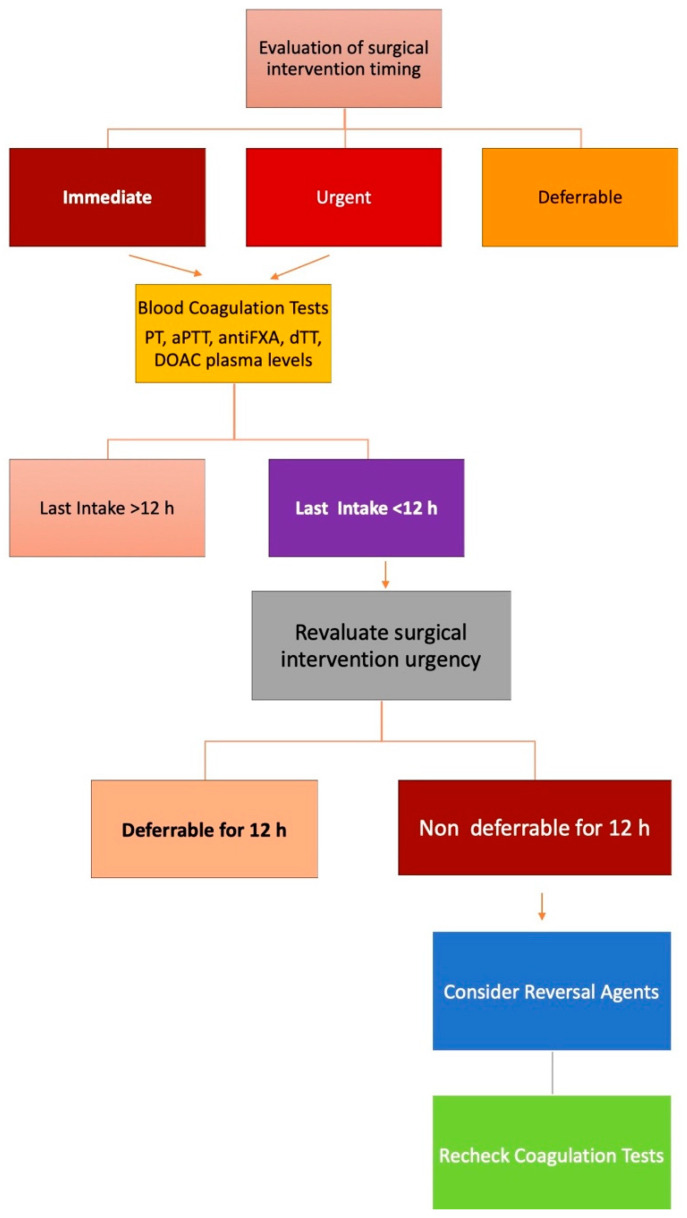
Management of patients treated with anticoagulant therapy needing surgical intervention. Legend: PT: prothrombin time;aPTT: activated partial thromboplastin time; anti FXA: anti-factor Xa;dTT: dilute thrombin time; DOACs: direct oral anticoagulants.

After a reversal of an oral anticoagulant, the main issue is to establish when an antithrombotic drug should be restarted. There is a lack of data from both randomized trials and real-world studies. A multidisciplinary team and close collaboration among different specialists are mandatory. Generally, when a clear indication of anticoagulation persists and the bleeding risk is not life-threatening, oral antithrombotic therapy should be restarted at the lowest effective dose [89]. In the following paragraphs, we will focus on the suggested strategy for patients needing a reversal agent for an unplanned surgery or during an MB under therapy with factor IIa, factor Xa inhibitors, and VKA inhibitors (Figure 2).

### 1.9. Inhibitor of VKAs (Warfarin)

In patients treated with VKA and needing urgent surgery or facing MB events, a few options are available to reverse VKA anticoagulation effects. Prothrombin complex concentrates (PCCs) are complexes of several clotting factors, including factors II, VII, IX, and X, and proteins C and S. PCCs are available as three - or four- factor complexes. Both can rapidly restore hemostasis in VKA patients by providing the necessary clotting factors. In a randomized control trial [90] in which patients with VKA treatment needing an emergency surgery (or invasive procedure) were randomized to receive either four-factor PCC or plasma, it was found that the administration of PCC significantly reduced the time to hemostasis compared to plasma infusion (median time to hemostasis of 2.5 h vs. 8.0 h). Furthermore, there were no significant differences in the incidence of thromboembolic events between the two groups. The authors concluded that PCC is an effective and safe reversal agent for warfarin. 

When PCC is unavailable, fresh frozen plasma (FFP) is a largely available alternative to reverse the effect of VKA [91]. FFP is a blood product that contains all clotting factors, including those affected by warfarin. The administration of FFP can restore hemostasis in patients taking warfarin by providing the necessary clotting factors, but it is aggravated by a non-neglectable rate of complications, such as allergic reactions [92]. Further disadvantages of FFP infusion include large infusion volume, with the consequent risk of fluid overload and infection risk [79]. Four-factor PCC represents the best option for a rapid reversal; those complexes should be administered throughout a stepwise approach based on INR (e.g., 25 U/kg if INR is 2–4.0; 35 U/kg if INR is 4–6.0; and 50 U/kg if INR is >6.0) [93]. Of note, since clotting factors have a short half-life, intravenous administration of vitamin K (5–10 mg) must be considered in cases where patients experience life-threatening bleeding during treatment with VKAs [79].

### 1.10. DOAC Non-Specific Reversal Agents 

When a patient in therapy with a DOAC needs urgent surgery or has MB, the first choice, whenever available, is a targeted reversal; the use of PCC is off-label in this situation. However, whenever a specific reversal agent is unavailable, PCCs could be considered to reverse the effect of DOACs. As those complexes contain coagulation factors, they can induce a non-specific reversal of DOACs [94]. Although they are currently used in clinical practice, data in patients with MB are limited and of low quality, and specific reversal agents should be preferred [79,95]. 

#### 1.10.1. Idarucizumab

Idarucizumab is a monoclonal antibody, the specific antidote for dabigatran (Table 3) [96]. This reversal agent has a very high affinity with dabigatran, forming indissoluble circulating complexes eliminated with renal metabolism. The effect of idarucizumab has been assessed in the Reversal Effects ofidarucizumab on Active Dabigatran (RE-VERS AD) study. In this study, patients were divided into group A (uncontrolled bleeding) and group B (urgent procedure). In the A group, the majority of patients had gastrointestinal (45%) and intracranial bleeding (33%). In contrast, in the group B, abdominal condition or infection (24%), fracture or septic arthritis (20%) and cardiovascular condition (18%) were the main causes leading to an urgent invasive procedure [97]. This study showed an 88–98% reversed effect of dabigatran (measured with ECT or TT) within a few minutes and a 100% reversed effect 4 h after a 5 mg intravenous administration. The administration was performed in two doses of 2.5 mg, given ≤15 min apart [97].In the bleeding group, the time to cessation of bleeding was 2.5 h. In contrast, in the surgical cohort, the time to initiate the procedure was 1.6 h, with a surgeon assessment of “normal” hemostasis during the procedure occurring in 93.4% of cases [97]. 

Although the pharmacological test did not show any prothrombotic activity of idarcuzimab, the RE-VERSE AD study showed thrombotic complications with a 30-day rate of 6.3% [98]. In this regard, the authors highlighted that mostthrombotic complications occurred days or weeks after idarucizumab administration, which exceeded the 45-minute half-time of this drug. Furthermore, in patients who experienced thrombotic events within 72 h, anticoagulation was not resumed, exposing patients to a prothrombotic state. 

Before the introduction of idarucizumab, the only available strategy to counteract dabigatran’s anticoagulant effect was drug removal with hemodialysis [99], which could still be useful in very selected patients, especially in the presence of chronic or acute kidney disease [100]. 

#### 1.10.2. Inhibitor of Factor Xa

Andexanet alfa is a recombinant modified factor Xa protein that is designed to bind and reverse the anticoagulant effects of any factor Xa inhibitors (Table 3). The drug has been found to be effective in patients with acute MB associated with the use of either rivaroxaban or apixaban. In the ANNEXA-4 study (phase 3b), after a 12 h period from the start of andexanet alfa infusion, more than 80% of the patients enrolled reached a good hemostasis status, and the bleeding was controlled [15]. However, in the study, some safety issues were raised regarding the high rates of ischemic events (ischemic stroke 4%, deep-vein thrombosis 4%, others 2%). It is important to highlight that patients treated with andexanet alfa are unresponsive to heparin, as it is also antagonized by this reversal agent.

In a recent study on 479 patients with acute MB (51% on apixaban, 37% on rivaroxaban, 8% on edoxaban, and 5% on enoxaparin), a good or excellent hemostatic efficacy was achieved in 80% of patients treated with andexanet alfa [15].

#### 1.10.3. FuturePerspectives

However, ciraparantag is an emerging compound with promising characteristics. It is described as a small, synthetic, water-soluble cation that can bind to both heparins and DOACs.To the best of our knowledge, only preliminary clinical data involving 107 healthy volunteers are presently available [101], and there remains a scarcity of randomized clinical trials investigating the efficacy of this drug.

## 2. Summary

The number of patients treated with DOACs has consistently increased over the years. Patients treated with anticoagulants have an increased risk of bleeding. Specific tests are now available to assess the plasma level of anticoagulant therapy. In patients treated with DOACs, specific reversal agents (idarucizumab and andexanet alfa) have been shown to be effective to counteract the anticoagulant effect.

## 3. Conclusions

DOACs have been shown to have a safe and effective profile and should be preferred to VKAs in most cases, excluding particular conditions such as rheumatic heart disease or mechanical heart valves [102]. However, some drawbacks have been described.

Patients diagnosed with thrombotic antiphospholipid syndrome who were randomly assigned to receive DOACs rather than VKAs exhibited an elevated risk of arterial thrombosis. Nevertheless, no notable variances were observed between patients randomized to DOACs versus VKAs concerning the risk of subsequent VTE or MB [103]. 

The development and clinical introduction of reversal agents for DOACs, such as idarucizumab for dabigatran and andexanet alfa for apixaban and rivaroxaban, have greatly facilitated the rapid antagonism of the anticoagulant effect of DOACs, significantly enhancing the management of bleeding in patients receiving DOAC therapy. However, despite these agents leading to the neutralization of the anticoagulant effect of DOACs in the majority of cases, the mortality rate in DOAC-related bleedings, particularly in ICH, remains high. Furthermore, significant limitations in using DOAC reversal agents in clinical practice persist, including the potential unavailability, the high costs, the difficulty of laboratory-specific tests, and concerns regarding potential paradoxical thromboembolic effects, particularly associated with andexanet. Further studies are needed in order to assess and compare the outcomes of using reversal agents.

## Figures and Tables

**Table 1:** Impact of DOACs on coagulation assays.

Coagulation Assays		Time to Obtain Test and Result Availability	Dabigatran	Rivaroxaban	Apixaban	Edoxaban
Routine coagulation assays	APTT	Usually within 1 h and 24/7	↑↑↑	↑↑ at C_max_	Slight or no change	↑ at C_max_
	PT/INR	Usually within 1 h and 24/7	↑ at C_max_	↑	Slight or no change	↑
Unspecific sensitive assays	TT	Usually within 1 h and 24/7	↑	Not applicable		
	ECA	Within hours and infrequently 24/7	↑			
Specific sensitive assays	dTT	Within hours and infrequently 24/7	↑			
	Chromogenic anti-Xa assays	Within hours and infrequently 24/7	Not applicable	↑		

Legend: aPTT: activated partial thromboplastin time; PT: prothrombin time; INR: international normalized ratio; TT: thrombin time; ECA: ecarin chromogenic assay; dTT: dilute thrombin time; C_max_:maximum concentration: C_max_: maximum concentration.↑ mildly increased; ↑↑ moderately increased; ↑↑↑ highly increased.

**Table 3:** Principal characteristics of DOAC specific reversal agents.

	Idarucizumab	Andexanet Alfa
Binding	Noncompetitive	Competitive
Target	Dabigatran	Xa inhibitors
Dose administration	5 mg (two infusions of 2.5 g/50 mL)	- Xa taken more than 8 h before: 400 mg IV bolus followed by continuous infusion of 4 mg/min up to 120 min (480 mg) - Xa taken less than 8 h before: 800 mg IV followed by continuous infusion of 8 mg/min (960 mg)
Anticoagulation effect	100% within 4 h	92% rivaroxaban 92% apixaban

Legend: h: hour, mg milligram, IV: intravenous.

## Data Availability

Data sharing is not applicable to this article as no new data were created or analyzed in this study.
